# Bedside adherence to clinical practice guidelines for enteral nutrition in critically ill patients receiving mechanical ventilation: a prospective, multi-centre, observational study

**DOI:** 10.1186/cc8915

**Published:** 2010-03-16

**Authors:** Jean-Pierre Quenot, Gaetan Plantefeve, Jean-Luc Baudel, Isabelle Camilatto, Emmanuelle Bertholet, Romain Cailliod, Jean Reignier, Jean-Philippe Rigaud

**Affiliations:** 1 Service de Réanimation Médicale, Bocage University Hospital, Boulevard de Lattre de Tassigny, 21079 Dijon, France; 2 Service de réanimation polyvalente, CH Victor Dupouy d'Argenteuil, 69 rue du Lieutenant Colonel Prudhon, 95107 Argenteuil, France; 3 Service de Réanimation Médicale, CHU de Saint-Antoine, 184 rue du faubourg Saint-Antoine, 75012 Paris, France; 4 Service de Réanimation Médicale, Hôpital Emile Muller, 20 Avenue du Docteur René Laennec, 68100 Mulhouse, France; 5 Service de Néonatologie, CHU de Lyon, 59 Boulevard Pinel, 69500 Bron, France; 6 Service de Biostatistiques et Informatique Médicale, Département d'Information Médicale, Bocage University Hospital, Boulevard de Lattre de Tassigny, 21079 Dijon, France; 7 Service de Réanimation polyvalente, CHD les Oudairies, 89925 La Roche sur Yon Cedex 09, France; 8 Service de Réanimation polyvalente, CH de Dieppe, Avenue Pasteur, 76202 Dieppe, France

## Abstract

**Introduction:**

The primary aim was to measure the amount of nutrients required, prescribed and actually administered in critically ill patients. Secondary aims were to assess adherence to clinical practice guidelines, and investigate factors leading to non-adherence.

**Methods:**

Observational, multicenter, prospective study, including 203 patients in a total of 19 intensive care units in France. The prescribed calorie supply was compared with the theoretical minimal required calorie intake (25 Kcal/Kg/day) and with the supply actually delivered to the patient to calculate the ratio of calories prescribed/required and the ratio of calories delivered/prescribed. Clinical factors suspected to influence enteral nutrition were analyzed by univariate and multivariate analysis.

**Results:**

The median ratio of prescribed/required calories per day was 43 [37-54] at day 1 and increased until day 7. From day 4 until the end of the study, the median ratio was > 80%. The median ratio of delivered/prescribed per day was > 80% for all 7 days from the start of enteral nutrition. Among the variables tested (hospital type, use of a local nutrition protocol, sedation, vasoactive drugs, number of interruptions of enteral nutrition and measurement of gastric residual volume), only measurement of residual volume was significant by univariate analysis. This was confirmed by multivariate analysis, where gastric residual volume measurement was the only variable independently associated with the ratio of delivered/prescribed calories (OR = 1.38; 95%CI, 1.12-2.10, p = .024).

**Conclusions:**

The translation of clinical research and recommendations for enteral nutrition into routine bedside practice in critically ill patients receiving mechanical ventilation was satisfactory, but could probably be improved with a multidisciplinary approach.

## Introduction

Nutritional support is now considered as a standard of care for intensive care unit (ICU) patients and has been the first-line choice for more than two decades [1]. The generally accepted goals of nutritional delivery in critically ill patients are to provide nutritional therapy consistent with the patient's condition, prevent nutrient deficiencies, avoid complications related to nutrition delivery, and improve patient outcome [2]. Most intensive care doctors aim to administer 25 Kcal/Kg/day, an energy target in line with recent recommendations [1-3]. Unfortunately, a number of factors render the provision of optimal enteral nutrition difficult, such as insufficient caloric targets, gastrointestinal dysfunction such as vomiting and diarrhea, repeated procedures and surgeries associated with interruption of enteral nutrition, feeding tube displacement, inadequate routine nursing procedures with delayed administration of the enteral feed, or premature enteral nutrition withdrawal [4-6]. The implementation of feeding protocols has been proposed as a strategy to optimize adequate delivery of nutritional support [7,8]. Despite a number of corrective measures proposed in recent years, exclusive enteral nutrition in ICU patients remains associated with nutritional deficiencies, and is correlated with impaired short- and long-term clinical outcomes [9,10]. To assess the translation of recommendations [1-3,7,8] into routine critical care, we measured the amount of nutrients required, prescribed and actually delivered in critically ill patients. Furthermore, we sought to identify the reasons for discrepancies between prescriptions and requirements, and between prescriptions and actual delivery of nutrition, through a prospective, observational, multicenter study. Preliminary results were presented at the 37^th ^Congress of the Société de Réanimation de langue Française (SRLF, French-speaking Society of Intensive Care) in Paris, in January 2009.

## Materials and methods

### Study design

An observational, prospective, multicentre study was conducted in 19 ICUs in France (see acknowledgements for complete list of participating centers). In early 2008, the Clinical and Epidemiology Research Commission (CERC) of the French-speaking Society for Intensive Care (SRLF) posted on its website a call for nurses to participate in a working group to evaluate practices in enteral nutrition and adherence to national guidelines published by the SRLF [1-3,7,8]. All 44 respondents, representing 24 French ICUs, were included in the working group, which also included four critical care physicians (members of the CERC). The study protocol (study variables, inclusion and exclusion criteria etc) was developed with the working group during a one-day meeting. No specific protocol for enteral nutrition was stipulated, in order to preserve the 'real world' nature of the observations. The members of the working group constituted the participating centers. As this observational study required no deviation from routine medical practice, institutional review board approval was not required. The study was approved by the Ethics Committee of the SRLF.

### Patient population

Over a period of two months (15 August to 15 October 2008) consecutive patients receiving mechanical ventilation and without contraindication to initiation of enteral nutrition (e.g., gastrointestinal bleeding, ileus, suspected perforation, abdominal surgery, prone positioning) or to insertion of a small-bore feeding tube (e.g. active variceal bleeding) were considered eligible for the study. Patients receiving non-invasive mechanical ventilation or parenteral nutrition were excluded. Decisions related to care, time of insertion, type of feeding tube, type of enteral formula, and use of prokinetic medication were guided by the multidisciplinary team caring for the patient. All patients received enteral nutrition via continuous infusion by a feeding pump. The amount of enteral nutrition delivered was quantified daily. Daily caloric intake was determined by multiplying the total amount of enteral nutrition delivered by the caloric content of the formula(s) and was recorded every morning.

A local protocol for enteral nutrition (no details available) previously existed and was applied in 12 ICUs, while only seven ICUs systematically measured gastric residual volume (GRV).

### Data collection

For each patient, the following data were recorded on admission: age, gender, body mass index (BMI = weight in Kg divided by height in meters squared), primary diagnosis and Simplified Acute Physiology Score (SAPS) II [11]. Prescriptions of sedation and vasoactive drugs were also recorded. The reasons for interruptions of enteral nutrition were recorded (weaning, radiology, emesis, diarrhea, problems with the small-bore feeding tube etc) for the seven days of the study period.

The duration of mechanical ventilation was also recorded.

Each day until day seven (or until patients were extubated, whichever came first), the amount of nutrients prescribed enterally and the amount of nutrients actually delivered to each patient was recorded by the nurses in each ICU. The optimal minimal calorie supply was set at 25 Kcal/Kg/day in accordance with current guidelines [1-3]. For obese patients (BMI >30 Kg/m^2^), optimal calorie intake was calculated for a theoretical weight corresponding to a BMI of 30 kg/m^2^. The Harris-Benedict equation adjusted for stress factors was not used in participating ICUs for calculation of required calories. Length of stay in the ICU and in-hospital, as well as mortality were also recorded.

### End points for enteral nutrition efficacy

The primary objective of this study was to calculate the ratio of prescribed to required calories, and the ratio of calories actually delivered to calories prescribed. The prescribed calorie supply was compared with the theoretical minimum required calorie intake (25 Kcal/Kg/day), and the calorie supply actually administered to the patient was compared with the prescribed amount. As a secondary endpoint, we analyzed factors likely to influence enteral nutrition and contribute to non-adherence to published guidelines for enteral nutrition.

### Data evaluation and quality control

All data except SAPS II and patient outcome were collected by the investigating nurses in each ICU. An independent research assistant entered data into a database using a specific computer program (Microsoft Excel, Microsoft Corp., Redmond, WA, USA). The program included reliability checks based on ranges for all data, and logical checks for inconsistencies and missing data. The members of the CERC carried out extensive data cleaning, and queries were addressed to the investigators for questionable or missing data.

### Statistical analysis

Continuous variables are reported as mean ± standard deviation or median (interquartile range). The median ratios of prescribed/required calories and delivered/prescribed calories were determined for the first seven days after the start of enteral nutrition or until the patient was extubated (whichever occurred first).

Clinical factors suspected to influence enteral nutrition (hospital type, use of a local nutrition protocol, sedation, vasoactive drugs, measured gastric residual volume and number of interruptions (divided into two classes <5 and >5)) were analyzed using the Mann-Whitney U test. Clinical factors suspected to influence the ratio of calories delivered/prescribed were analyzed by multivariate logistic regression. Variables associated with the ratio of delivered/prescribed calories by univariate analysis (*P *< 0.10) were entered into a stepwise logistic regression.

A *P *value less than 0.05 was considered significant. Statistical analyses were performed using SAS v 8.2 software (SAS Institute, Cary, NC, USA).

## Results

### Characteristics of study population

A total of 203 patients were included in the study (Table 1). Mean age was 62 ± 18 years; 134 (66%) were men. Mean SAPS II score on ICU admission was 53 ± 18 points. Mean BMI was 27 ± 8 Kg/m^2^. The participating ICUs comprised university and/or regional hospitals (n = 10, 52%), and general (non academic) hospitals (n = 9, 48%). There were 6 (31%) mixed medico-surgical and 13 (69%) medical ICUs. The mean number of beds in ICUs was 14 ± 3.

**Table 1:** Patient characteristics

Number of patients	203
Hospital type (n)	
Academic	89
Community	114
Age (years)	62 ± 18
Gender (male/female)	134/69
SAPS II (points)	53 ± 18
Body mass index (kg/m^2^)	27 ± 8
Primary diagnosis, n (%)	
Respiratory	65 (32)
Cardiovascular	11 (5)
Neurologic	54 (27)
Renal	4 (2)
Post surgical	7 (3)
Septic shock	28 (14)
Traumatologic	10 (5)
Burns	1 (0.5)
Other	23 (11)
Mechanical ventilation (days)	12 ± 9
Length of ICU stay (days)	15 ± 13
Length of hospital stay (days)	28 ± 19
ICU mortality, n (%)	50 (25)
In-hospital mortality, n (%)	65 (32)

Continuous variables are reported as mean ± standard deviation and categorical variables as number of patients (percent)

ICU: intensive care unit; SAPS II, simplified acute physiologic score II.

### Primary endpoint: calories prescribed, required and actually delivered

The median ratio of prescribed/required calories per day was 43 (37 to 54) on day one and increased until day seven (Table 2). From day four until the end of the study, the median ratio was more than 80%. The analysis concerned all 203 patients on day one, and decreased to 110 patients on day seven, due to interruptions to enteral nutrition and/or extubation in some patients.

**Table 2:** Ratio of prescribed to required calories, ratio of delivered to prescribed calories and ratio of delivered to required calories per day

Day	Number of patients on each day	% of prescribed/required	% of delivered/prescribed	% of delivered/required
1	203	43 (37-54)	85 (77-92)	36 (29-44)
2	189	67 (59-76)	85 (76-91)	57 (48-65)
3	166	73 (65-88)	90 (80-98)	66 (58-73)
4	148	80 (71-91)	90 (79-97)	72 (65-80)
5	130	86 (78-93)	93 (82-101)	80 (71-88)
6	116	88 (79-95)	90 (79-99)	79 (70-86)

7	110	87 (78-94)	93 (81-102)	81 (73-88)

Values are expressed as median (interquartile range).

The median ratio of delivered/prescribed per day was more than 80% over the seven days from the start of enteral nutrition.

### Secondary endpoint: factors suspected to influence enteral nutrition

We evaluated by univariate analysis the following variables, considered likely to influence enteral nutrition, and contribute to non-adherence to feeding guidelines: hospital type, use of a local nutrition protocol, sedation, vasoactive drugs, number of interruptions, and measurement of GRV (Table 3). Among the variables tested, only the systematic measurement of GRV was significantly associated by univariate analysis with the mean ratio of prescribed/required and delivered/prescribed calories: when GRV was not measured, there was a significantly higher mean ratio of prescribed/required and delivered/prescribed calories (*P *< 0.05). This was confirmed by multivariate analysis, where GRV measurement was the only variable independently associated with the ratio of delivered/prescribed calories (odd ratio = 1.38; 95% confidence interval = 1.12 to 2.10, *P *= 0.024). In practice, when GRV is measured, there is a 38% increase in the risk of having a low ratio of delivered/prescribed calories.

**Table 3:** Variables influencing the total ratio of delivered to prescribed calories over the seven-day study period by univariate analysis

Variable	Number of patients	% prescribed/required	*P *value	% delivered/prescribed	*P *value
Hospital type			0.91		0.67
Academic	89	70 (59-78)		86 (79-97)	
Community	114	72 (63-80)		87 (80-97)	
Local protocol			0.38		0.94
Yes	137	73 (65-79)		88 (81-100)	
No	66	66 (59-73)		84 (79-98)	
Sedation			0.86		0.03
Yes	150	66 (58-78)		89 (82-101)	
No	53	62 (54-71)		80 (71-87)	
Vasoactive drugs			0.32		0.77
Yes	102	70 (59-79)		88 (81-99)	
No	101	72 (61-80)		86 (79-92)	
GRV measured			0.002		0.01
Yes	135	68 (59-77)		83 (76-89)	
No	68	77 (69-84)		95 (90-104)	
Number of interruptions					
<5	180	71 (63-79)	0.42	71 (66-78)	0.08
>5	23	69 (58-75)		65 (59-72)	

Values are expressed as median (interquartile range); GRV: gastric residual volume

## Discussion

This is the first multicenter study to assess the level of bedside adherence to clinical practice guidelines for enteral nutrition in critically ill patients receiving mechanical ventilation further to the publication of recent guidelines [1-3].

The main finding of our study is a good ratio of calories actually delivered/prescribed (>80%) and calories prescribed/required (>80%), notably after 72 hours. These results are better than those observed in recent studies in similar populations [5-8,12,13]. We observed a satisfactory ratio of delivered/prescribed calories, exceeding 80%, indicating that in general, medical prescriptions are accurately applied by the ICU team over the first seven days.

The main objective of nutrition in critical care is to obtain a calorie content of 25 to 35 Kcal/Kg/day at two to three days [1-3]. The amount of calories is based on measurement of oxygen consumption (indirect calorimetry) as the reference method, but this requires costly equipment and technical skills that are not widely available, as well as being time-consuming [14]. Another method is the assessment of resting energy expenditure using the Harris-Benedict formula [15], which is a simple formula that takes into account the patient's weight, height, age, and gender.

Previous reports have shown that the calorie supply prescribed and that actually delivered are often below the patients' theoretical needs, because of late initiation, unjustified or excessively long interruptions, diagnostic procedures, airway management, mechanical problems, and failure to reinstill GRV samples [5,16,17]. The tolerability of enteral nutrition is sometimes poor, especially in case of treatment with vasoactive drugs, shock, or sedation, or in case of vomiting, repeated interruption of enteral feeding, or upper digestive intolerance [13,17,18]. In our study, the only factor that significantly influenced the prescribed calories and the level of actually delivered calories by univariate analysis was the measurement of GRV. This could be explained by the fact that GRV measurement by ICU nurses is either systematic (i.e. stipulated by local protocol), particularly at the time of initiation of enteral nutrition; or else applied in case of regurgitation, which hinders the achievement of daily calorie intake goals. In this case, the nurses tend to lower the flow rate, or even stop enteral nutrition altogether.

In one recent report, immediate introduction of the optimal dose of enteral nutrition was associated with significantly more episodes of GRV of more than 300 ml and with a trend towards more frequent use of prokinetic agents [19]. The impact of GRV on the risk of serious adverse events is controversial, and controversy persists regarding the threshold predictive of unfavourable outcome (about 250 ml) [20]. A recent study has shown a non-consistent relation between aspiration and GRVs [21]. The role of gastrointestinal dysfunction might have been reduced by the fact that the decision to start, increase, reduce, or stop enteral nutrition was made by the physician according to the patient's clinical condition, especially the gastrointestinal tract status (vomiting, diarrhea, or abdominal pain or distension). Our study was not designed to evaluate gastrointestinal tolerance to enteral feeding, because such an evaluation would have required a standardized protocol for enteral nutrition to be applied in all participating centers.

Interestingly, we observed a significantly higher ratio of delivered/prescribed calories in sedated patients. This could be explained by the fact that physicians tend to prescribe less enteral nutrition because of the risk of regurgitation among these patients, and thus, ICU nurses would generally have proceeded as usual in accordance with their standard practice or as stipulated in any local protocol.

Recent evidence suggests that even with the best intensive educational programs to increase compliance with enteral nutrition guidelines, patients receive only 50% of the prescribed requirements [22].

In our study, the existence of a local protocol had no effect on the total percentage of calories delivered or prescribed, perhaps because published guidelines are simple and easily applicable [1-3]. Clinical trials to assess interventions and outcomes in enteral nutrition may not be applicable to everyday practice, given that delivery of prescribed enteral nutrition is commonly incomplete. Therefore, we believe that the results of this 'real world' study are a powerful tool to inform about the processes used to feed patients [23].

Most procedure and radiological studies require the patient to be supine, a requirement that interrupts enteral nutrition because of the increased risk of aspiration. Together, procedures and radiological studies accounted for 13% of the interruptions in enteral nutrition [13].

We observed in our study a discrepancy between required and prescribed calories, which can most probably be explained by under prescription on the part of the physicians. Insufficient information, notably absence of BMI data at admission, likely led to sub-optimal prescription.

In our study, we did not assess the effect of enteral nutrition on patient outcome. Few studies have demonstrated the capacity of enteral nutrition to reduce infectious complications, improve nutritional endpoints, or decrease mortality [1,23]. A recent study [4] demonstrated that although successful implementation of the guidelines resulted in a significant change in practice, it did not lead to reduced hospital mortality in critically ill patients.

### Study limitations

There are several limitations associated with the methods used in this study. The protocol used in participating ICUs was not stipulated in detail, notably as regards use of the Harris-Benedict formula [15], prokinetic medication or measurement of GRV. Also, local protocols were generally based on the same French and international recommendations [1-3]. The results would likely have been significantly different if a reference level for theoretical calorie requirements above 25 Kcal/kg/day had been used. It should be noted that there was a considerable reduction (about 50%) in the number of participants after day three, which undoubtedly reduces the power of this study and the results should be interpreted with care. Also, it should be noted that we were unable to calculate the caloric uptake contained in infusions or the lipid content of propofol infusions.

Furthermore, the patient population was predominantly non-surgical, and any conclusions are restricted to this population and the results of this study cannot be extrapolated to other patient types or all other ICUs in France, because the patient populations may be significantly different in other centers.

Finally, although multivariate analysis was performed, its results should be interpreted with caution, because this was an observational study, and it is impossible to take into account all confounding factors.

## Conclusions

This study is in line with efforts at European level to evaluate professional practices, and quantify the differences between what is recommended in clinical guidelines and/or the medical literature, and what actually happens in daily routine practice at the bedside.

We proposed a multidisciplinary approach to nutritional support including nurses, dieticians, and pharmacists, with regular training of medical staff involved in nutrition support prescription and delivery. A comprehensive review of routine practice in ICUs might help to achieve optimal nutrition care for critically ill patients.

The translation of clinical research and recommendations for enteral nutrition into routine critical care at the bedside in critically ill patients receiving mechanical ventilation was satisfactory, but could likely be improved with the use of a multidisciplinary approach.

## Key messages

• In patients receiving enteral nutrition, the calorie supply prescribed and that actually delivered are often below the patients' theoretical needs.

• We performed an observational, multicenter study in a representative sample of ICUs to evaluate theoretical calorie requirements, calories prescribed, and actual calories delivered in ICU patients, in light of guidelines for enteral nutrition.

• We observed a good ratio of calories actually delivered/prescribed (>80%) and calories prescribed/required (>80%), notably after 72 hours, indicating that in general, medical prescriptions are accurately applied by the ICU team over the first seven days.

• In our study, the only factor that significantly influenced the prescribed calories and the level of actually delivered calories by univariate analysis was the measurement of GRV. This was confirmed by multivariate analysis, where GRV measurement was the only variable independently associated with the ratio of delivered/prescribed calories.

## Abbreviations

BMI: body mass index; CERC: Clinical and Epidemiology Research Commission; GRV: gastric residual volume; ICU: intensive care unit; SAPS: Simplified Acute Physiology Score; SRLF: Société de Réanimation de langue Française.

## Competing interests

The authors declare that they have no competing interests.

## Authors' contributions

JPQ was involved in study conception and design, acquisition of data, analysis and interpretation of data and drafting and critical revision of the manuscript. GP was involved in study conception and design and acquisition of data. JLB was involved in study conception and design and acquisition of data. RC was involved in analysis and interpretation of data and acquisition of data. JPR was involved in analysis and interpretation of data, acquisition of data, and drafting and critical revision of the manuscript. JR was involved in acquisition of data and drafting and critical revision of the manuscript. All authors read and approved the final version of the manuscript.
